# Improvement of online monitoring of drinking water quality for the city of Prague and the surrounding areas

**DOI:** 10.1007/s10661-021-09534-9

**Published:** 2021-10-30

**Authors:** Přemysl Soldán

**Affiliations:** grid.438481.2 0000 0001 0940 8879 T. G. Masaryk Water Research Institute, Macharova 5, 702 00 Ostrava, Czech Republic

**Keywords:** Drinking water, Water treatment, Accidental pollution, Continuous biological monitoring

## Abstract

In the article, a new method of continuous monitoring of the biological quality of raw and treated waters at the Želivka Water Treatment Plant is suggested and assessed. This water treatment plant is one of the largest water treatment plants in Europe and the largest water treatment plant in the Czech Republic with a maximum projected peak output of 7 m^3^.s^−1^ and current output of around 3 m^3^.s^−1^ of drinking water. It is the largest water treatment plant for Prague which is the capital city of the Czech Republic. Additionally, this water treatment plant also supplies drinking water to the Central Bohemia and Vysočina regions. The main intention of the research was to suggest a new system of monitoring, which can guarantee a more reliable continuous control of the safe drinking water supply for the city of Prague and the surrounding area. The suggested method represents a completely new approach to monitoring the biological quality of drinking water in the Czech Republic using the DaphTox apparatus, only two of which exist in the Czech Republic. The article describes the experience and knowledge gained during the operation of such a monitoring system, including a description of the necessary measures to ensure its proper operation with a focus on the pre-treatment of raw and chlorinated waters. Recommended simple pre-treatment methods secure optimal living conditions for monitoring organisms and have no impact on the original biological quality of monitored water which is a necessary condition for proper monitoring of biological quality.

## Introduction

“Clean water—a healthy city” is not only a generally valid statement but also the name of the project, which was solved within the framework of the Operational Programme Prague—Growth Pole of the Czech Republic. This programme was financed by the European Structural Funds and the European Regional Development Fund.

The focus of the project was an improvement in the protection of water sources for the population of Prague and on raising awareness of potential problems. The main research goals were:

- Reduction of the potential health risks by more accurate identification of possible contamination of drinking and surface water
- Increase in the level of knowledge about the causes and consequences of pollution of surface water and wastewater
- Support of the introduction of new environmentally favourable technologies and practices

Due to the complexity of several interrelated issues, e.g. contamination of drinking water reservoir and river sediments, a survey of sources of pollution in the river basin, the use of activated carbon sorbents for water treatment, improvement of early warning system at the water treatment plant and presence of selected pollutants in Prague wastewaters, the project consisted of four relatively independent parts called concepts. The following text describes the experience and results gained in the concept which dealt with the improvement of the early warning system at the Želivka Water Treatment Plant.

### The Želivka Water Treatment Plant

The Želivka Water Treatment Plant (ZWTP) and its subsidiary company the Želivská Provozní are water companies that participate in the management and operation of the Central Bohemian Water System.

The treatment plant was put into operation in 1972. Raw water is supplied to the treatment plant from the Švihov reservoir, which with its water volume of 266.5 million m^3^ is the largest drinking water reservoir not only in the Czech Republic but also in Central Europe. The quality of water in this reservoir is endangered in the long-term period mainly by eutrophication, pesticide pollution and erosion (Liška et al., 2016; Mičaník et al., 2020).

The basic technology of water treatment used here is coagulation filtration with dosing of aluminium sulphate. Produced drinking water is hygienically protected with ozone and chlorine. Due to the risk of pollution of water from the reservoir, which was mentioned in the previous paragraph, the construction of filtration units with granulated activated carbon (GAC) is currently underway at the treatment plant (Tušil, 2016).

With a maximum projected peak output of 7 m^3^.s^−1^ of drinking water and current output of around 3 m^3^.s^−1^ of drinking water, the ZWTP is one of the largest water treatment plants in Europe and the largest water treatment plant in the Czech Republic. It is also the most significant water treatment plant for the capital city of Prague. The transport of drinking water to Prague is ensured by a gallery feeder with a length of 51.97 km to the Jesenice I reservoir near the village of Vestec. The share of the ZWTP in the supply of drinking water to Prague is around 74% (according to the data of the Prague Water Supply and Sewerage System Company). The ZWTP also supplies drinking water to the Central Bohemian Region and the Vysočina Region. This represents supply to about 1.2 million inhabitants in total.

The risk of sudden contamination of water with hazardous substances is, of course, relatively high for surface sources of raw water. In the case of the ZWTP, raw water is sourced from the surface Švihov drinking water reservoir. At present, the risk of unwanted contamination is increased by the risk of targeted criminal or terrorist activity.

A sudden deterioration in water quality occurs completely unpredictably, so it is necessary to build an early warning system in treatment plants, which would quickly and especially reliably detect changes in the quality of raw water to a high degree of sensitivity.

### Early warning system

To ensure the safety of production, it is necessary to have real-time information on the quality of raw water flowing into the treatment plant and the water discharged from the treatment plant into the water supply network. Because the treatment of drinking water is a continuous process, continuous monitoring is necessary. Joint Research Centre prepared a document practical guidelines on the requirements of a continuous online water quality monitoring system in drinking water supply systems which complexly describes this problem (Carmi & Theocharidou, 2019).

Routine continuous monitoring of surface waters in the Czech Republic includes monitoring of selected physicochemical parameters (pH, dissolved oxygen content, conductivity, temperature, UV absorbance at 254 nm). These data are insufficient to assess the possible adverse biological properties of water, in particular those caused by the toxic effects of pollution (Soldán, 2011).

Other highly specialized chemical analyses also do not meet the needs of the early warning system. They cannot be performed in a continuous mode, so there is a real risk that a wave of water contamination might not be detected. Moreover, these analyses are designed to target a specific substance, often determining concentrations to high degrees of accuracy and precision, rather than to screen for unknown potential contaminants. And last but not least, even very accurate data on particular contaminants present in water cannot be used to predict the final biological impact The resultant biological effects are determined by the physiological availability of these substances and are also fundamentally affected by the interaction of all substances contained in water.

The needs of the early warning system are therefore best met by continuous biological monitoring, where selected monitoring organisms are continuously exposed to the monitored waters and the assessment of biological properties of these waters is based on evaluating the response of these organisms to the overall composition of these waters.

### Online monitoring of water quality at water treatment plants of the Czech Republic

For the above-mentioned reasons, most water supply companies in the Czech Republic have introduced continuous biological monitoring by observing the response of rainbow trout to the quality of raw water flowing through fish tanks in which the organisms are located.

The use of rainbow trout for monitoring in water treatment plants in the Czech Republic is mainly dealt with by fish experts from the University of South Bohemia (Randák et al., 2011). However, these authors focus primarily on histopathological examinations of monitoring fish and analyses of pollutant content in fish tissues, which are indicators of long-term effects of monitored waters on fish, rather than of short-term fluctuations in biological water quality, as would be required for an early warning system.

Continuous monitoring for an early warning system is also operated at the ZWTP. At this treatment plant, trout are placed in two glass tanks through which raw water from the Švihov drinking water reservoir flows. The fish are constantly monitored by a camera, and their reaction is evaluated by experienced employees of the treatment plant. The surveillance camera is located approximately 5 m away from the tanks, which allows a comprehensive view of the tanks, but this distance reduces the “sensitivity” of the reading.

An on-the-spot investigation revealed that the fish in both tanks were in good health and showed significant weight gains, probably due to high feed intake. Observed fish size and the density of them in both tanks were quite high. This can negatively affect the “sensitivity” of detection of non-standard behaviour of individuals in the event of unsatisfactory biological water quality. The higher density of fish stocks can also lead to an increase in the aggressiveness of monitoring fish and to their mutual attacks (Dunaj n.d.)

Biological monitoring is currently applied only at the raw water inlet to the treatment plant, not at the outlet of drinking water. Thus, the biological quality of the water discharged to the water supply network after the treatment process is not monitored, although the treatment process could potentially lead to contamination by reaction intermediates with biological impact.

According to the recommendations of the US Environmental Protection Agency (Hasan et al., 2005), any comprehensive biological early warning system (BEWS), which is based on the evaluation of the reactions of living organisms, should meet several basic following requirements:

- It must provide a rapid response.
- It must detect a wide range of different contaminants, but at the same time maintain sufficient sensitivity.
- It must work as an automated system that allows remote control.

Any BEWS that does not meet these three basic characteristics is not considered an effective warning system.

The current system of monitoring the quality of water flowing into ZWTP does not meet some of these requirements, revealing shortcomings that significantly affect its detection ability and thus the overall level of control of biological properties of surface waters intended for the production of drinking water. This project aimed to introduce a new continuous method of monitoring the biological properties of water, which will significantly increase the efficiency, sensitivity and operability of the entire monitoring system of the treatment plant compared to the currently used method. At the same time, this continuous monitoring should be able to capture the possible negative effects of water treatment technology on its biological properties.

### Continuous biological monitoring devices in water treatment plants

The stated goals can be achieved by introducing the use of devices for biological monitoring, which have a continuous computer evaluation of the reaction of monitoring organisms, combined with automatic notification of a significant change in the quality of monitored waters.

The basic idea of using automatic biological sensors to monitor water quality was first expressed in the early 1970s (Gunatilaka & Diehl, 2001). Since then, there have been significant developments in this area. There are many companies worldwide that commercially offer devices for performing automated continuous biological monitoring.

These devices are used not only to monitor water quality in river basins, but also for water treatment plants (Storey et al., 2011). As an example, we present the Stakcin Water Treatment Plant in Slovakia (Bratská & Riganová n.d.; Dunaj n.d.). Two types of devices are installed on the raw water supply at this treatment plant—the FISH monitor of a German company Kerren Umwelt Technik, which uses rainbow trout as a monitoring organism, and the MOSSELMONITOR of a Dutch company Delta Consult, which uses molluscs *Unio pictorum* and *Unio tumidus* as a monitoring organism. Both devices work continuously with the automatic evaluation of the biological status of the monitored waters and triggering an alarm in the case of exceeding the limit values. In the event of an undesirable change in the quality of raw water, the system makes it possible to proceed quickly to shut down the treatment process, determine the causes of this change and take the necessary measures. The authors found the monitoring systems very effective, especially in terms of the high sensitivity of the devices and the speed of reaction to changes in the biological quality of water.

## Materials and methods

In the case of the ZWTP, we decided to use DaphTox devices produced by bbe Moldaenke (Germany) at the inlet and outlet of water from the treatment plant. The selection of this type of monitoring device depended on the following criteria:

- Device sensitivity
- Ease of use
- Level of verification by the routine operation

Selected monitoring organisms and the method of evaluation of their reactions determine the sensitivity of the device. In the case of the DaphTox apparatus, daphnids of *Daphnia magna* species are used as monitoring organisms. These organisms are highly sensitive to a wide range of pollutants. It is also important for the case of monitoring the quality of water from the reservoir that daphnids are very sensitive to the impact of toxins produced by cyanobacteria. The risk of water contamination with such substances is very high in the summer period. Many authors (Agrawal et al., 2012; Blanchette & Haney, 2002; Bownik, 2013; Ferrão-Filho & Azevedo 2003; Herrera et al., 2015; Schmidt et al., 2014; Vasconcelos, 2001; Zanchett & Oliveira-Filho, 2013) consider these organisms to be the most suitable for toxicity testing of cyanobacterial toxins due to their high sensitivity to the given substances. In contrast, trout (and fish generally) show very low sensitivity to these toxins, with even high concentrations leading to no discernable hepatopancreatic damage.

The evaluation of the response of daphnids used for monitoring in DaphTox devices already includes changes in behaviour, i.e. possible non-lethal negative effects of water pollution.

**Fig. 1:**
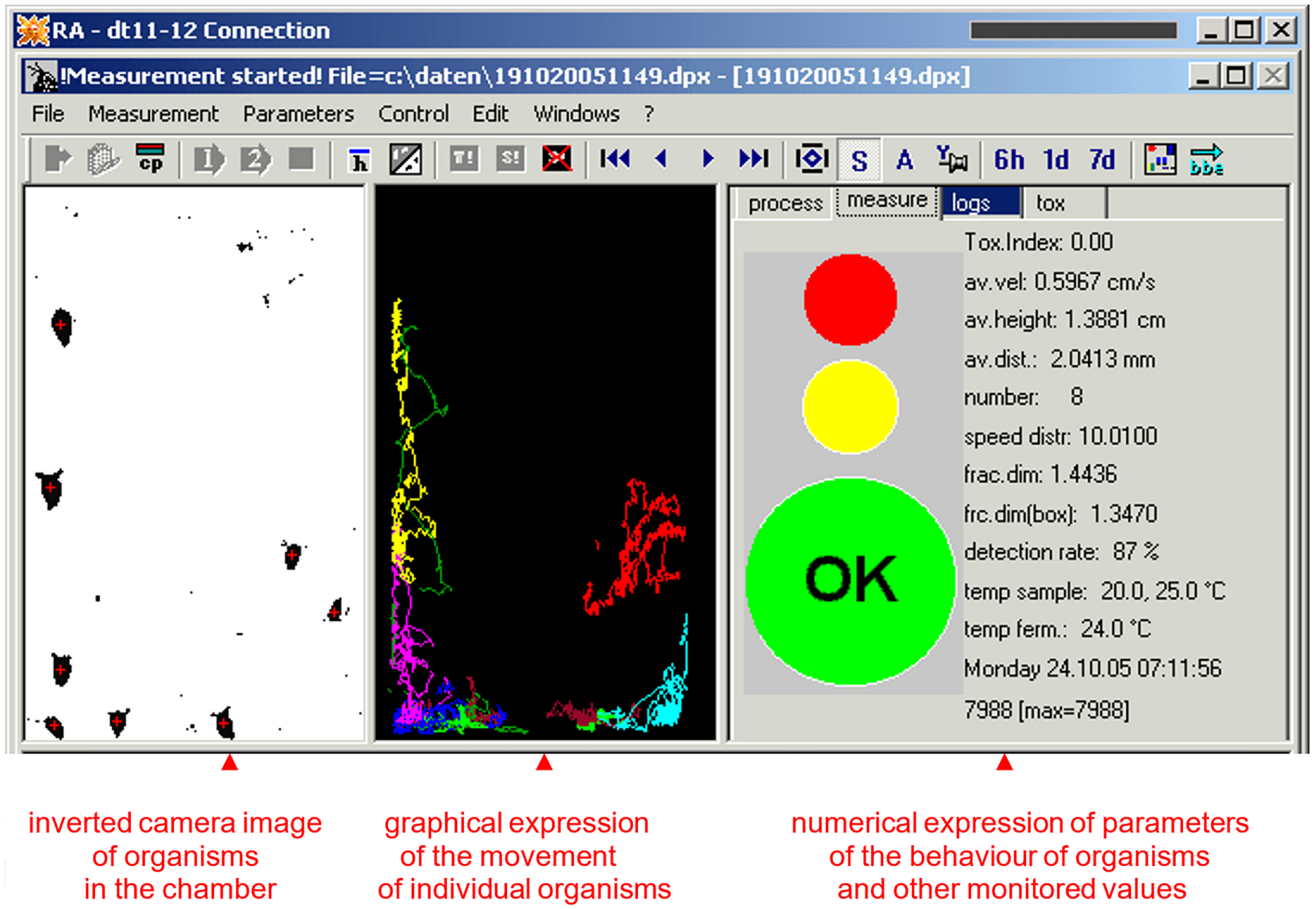
Camera image and its conversion into graphic and numerical form

The movement of organisms in the measuring cell is observed by a CCD camera. Behavioural changes are continuously evaluated by an integrated computer. Specialized software developed by bbe Moldaenke converts the image record into a graphic and then a numerical form (see Fig. 1).

**Fig. 2:**
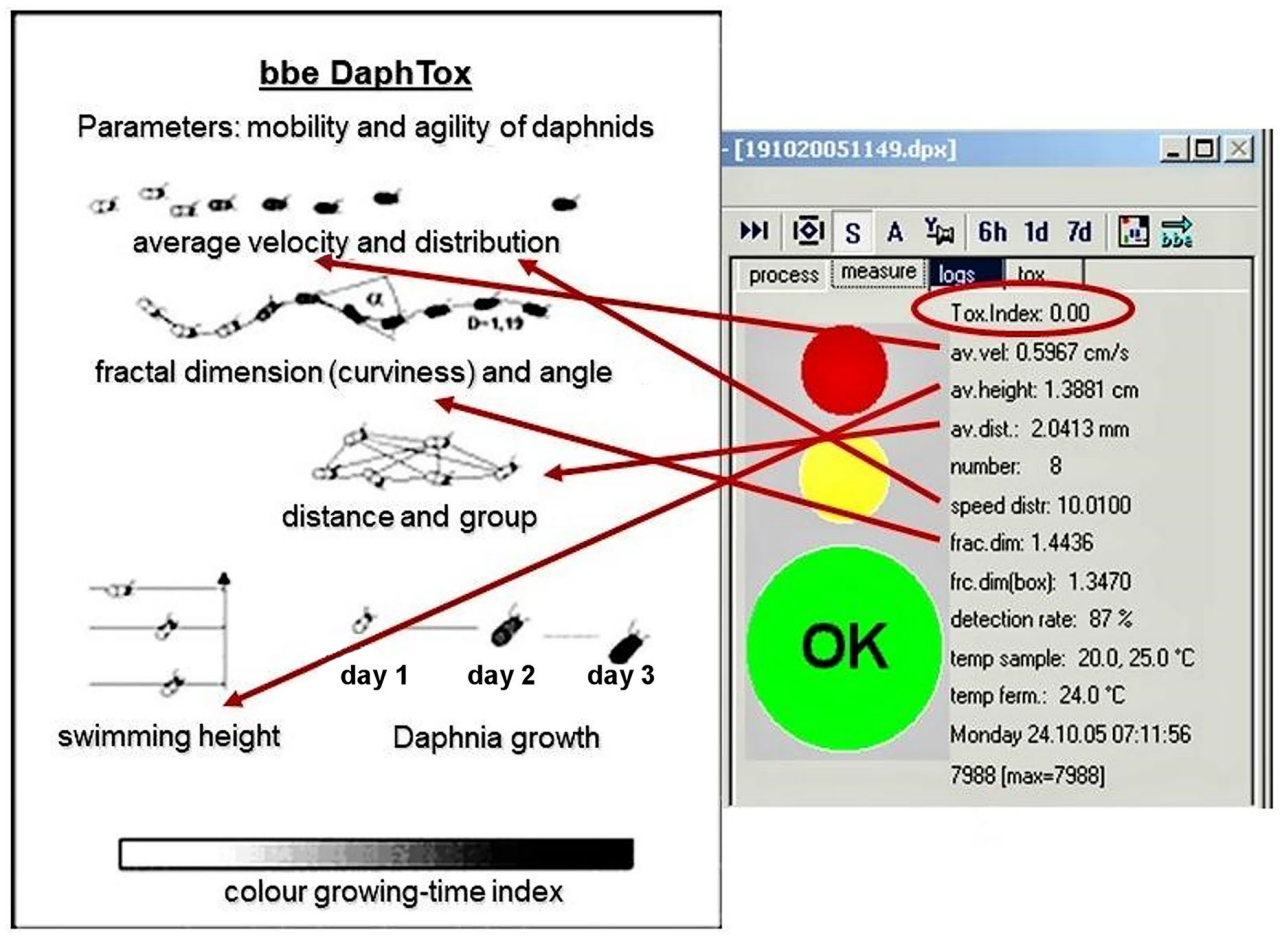
Evaluation of toxic index based on the assessment of various behavioural parameters of monitoring organisms (parameter description from bbe Moldaenke materials)

The assessment of animal behaviour is based on some calculated parameters, which take into account, for example, the average speed of movement of organisms, their position in the chamber and also their mortality (Lechelt et al., 2000). The so-called toxicity index, with values from 0 to 10, is determined from a series of data (see Fig. 2). Based on its value, a warning or alarm is triggered.

The device can be monitored online on the Internet from any computer or mobile device equipped with specialized software. This makes it possible to obtain current information on the biological quality status of water in the monitored profile from anywhere and at any time.

The operation of the device does not require specialized knowledge and can be fully ensured after proper training.

The level of verification of DaphTox devices by routine use is very extensive. In addition to installation at a wide range of monitoring stations located on rivers within different European river basins, these devices are also used worldwide to monitor water quality in the water and food industries (see Table 1 for a user overview provided by the device manufacturer).

**Table 1:** Use of DaphTox devices in the waterworks and food industry (bbe Moldaenke data)

Country	Company/enterprise	Application	From	Type of water
Brazil	HEINEKEN Brasil Jacaref	Beer brewing	2011	River water
Germany	Stadtwerke Konstanz	Drinking water production	2005	Lake water
	Warsteiner	Beer brewing	2013	Groundwater
	Zweckverband Bodensee-Wasserversorgung Sipplingen Lake Constance Water Supply Association – BWV	Drinking water production	2009	Lake water
Israel	Mekorot Water Co. Nazareth – Illit National Water Company	Drinking water production	2008	Unknown
Netherlands	Het Waterlaboratorium Nieuwegein	Monitoring of drinking water	2007–2009	River water
	WML Maastricht	Drinking water production	2009	River water
Switzerland	Zurich Water Company Zurich	Drinking water production	2013	Groundwater
	IWB/Industrielle Werke Basel Water and Energy Company	Water quality monitoring	2011	River water
USA	Salt Lake City Water Works	Drinking water production	2001–2002	Groundwater
Ukraine	The Desna Water Treatment Plant	Drinking water production	2015	Groundwater

### Installation of equipment and introduction of breeding of monitoring organisms

In the preparatory phase, consultations were held with the treatment plant operator, and on-site investigations were carried out, focusing on the technical solution for connecting the equipment to the raw and treated water supply. Then monitoring devices were installed at the water treatment plant.

The staff of the treatment plant very actively cooperated. They provided premises for the installation of devices, for the breeding of monitoring organisms (daphnids) and the cultivation of fodder planktonic algae, made necessary arrangements for pipeage of both types of monitored water to the monitoring room and also cooperated in making the online connection of devices to the Internet.

We decided to use devices of two generations—DaphTox I and DaphTox II. Their suitability for the needs of continuous monitoring of biological properties of water has been proven in a long trial operation at river monitoring stations in the Odra river basin (Soldán, 2011). Despite this fact, their proper function was verified by preliminary thorough laboratory testing.

**Fig. 3:**
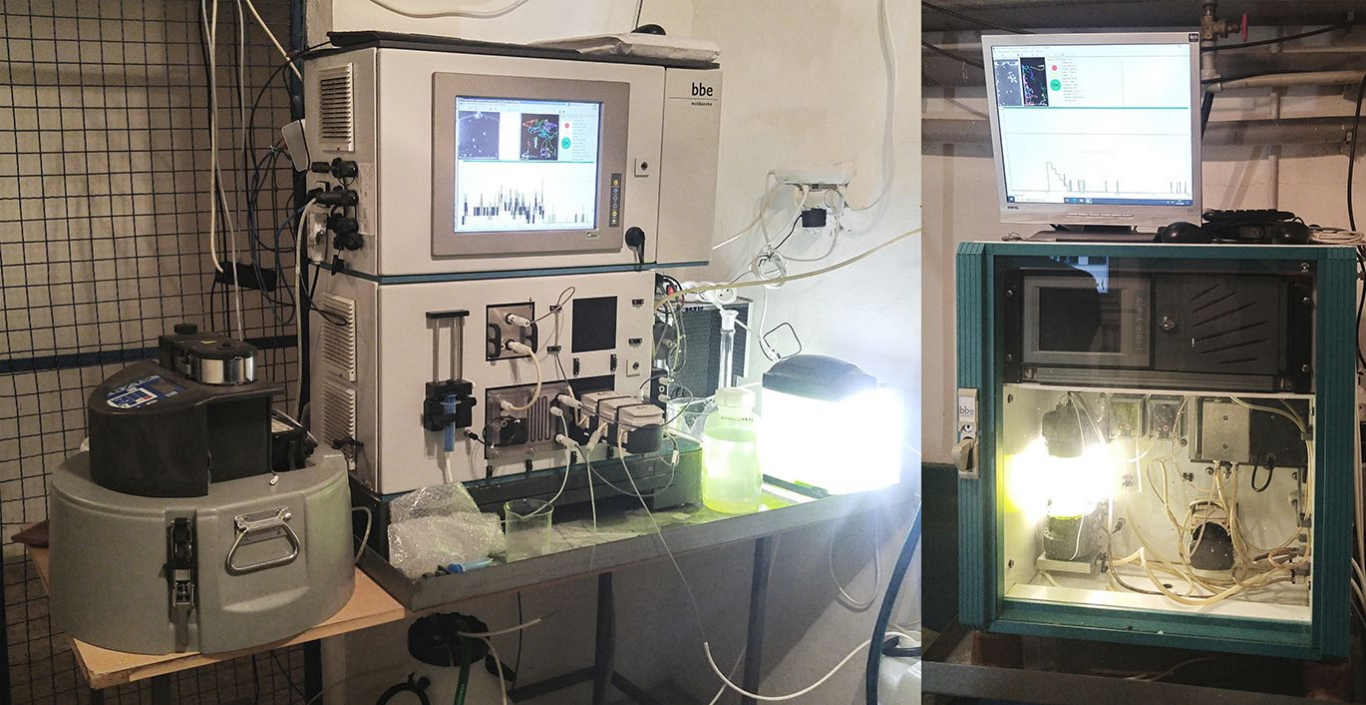
Monitoring devices installed at the Želivka Water Treatment Plant (left, DaphTox II; right, DaphTox I)

After these tests and implementation of the necessary technical adaptations in the monitoring room, these devices were installed in the water treatment plant (Fig. 3). At the same time, the breeding of monitoring organisms and the cultivation of algae was started. By setting up a connection via the Internet, online monitoring of the response of monitoring organisms was also started.

Before the start of regular operation, toxicological tests were performed on the premises of the treatment plant, aimed at detecting possible negative biological effects of the monitored types of water on monitoring organisms, i.e. *Daphnia magna*.

The effects of raw water, water after treatment and, for comparison, also standardized dilution water were verified by standardized acute toxicity tests (ISO 6341:2012, 2012). While the standardized dilution water did not have any negative effects at the exposure time of 48 h, the raw water already harmed the monitoring organism to a limited extent. The rapid death of 100% of the organisms occurred in the tested waters undergoing the treatment process. Verification in the DaphTox monitoring device fully corresponded to these results, when during the test monitoring the death of the monitoring organisms occurred to the extent corresponding to the results of the standardized tests. Solutions to these problems are described below.

### Pre-treatment of raw water

In the case of raw water, we initially concluded that it probably has different biological properties from the standard dilution water in which the monitoring organisms were originally bred. To adapt the organisms to this water, we started the breeding of them in raw water. The assumption was that newborn generations raised in raw water should be adapted to this medium. However, this step did not bring the desired result—the death of monitoring organisms in DaphTox also occurred in newly bred individuals, although they showed full adaptation to raw water (normal reproduction and sufficient production of new organisms). We concluded that elevated mortality of daphnids is possibly caused by the low concentration of dissolved oxygen in the monitored raw water. This water is taken from greater depths of the drinking water reservoir, where the concentration of dissolved oxygen decreases significantly. This hypothesis was supported by the results of measurements performed by the laboratories of the treatment plant. These results showed that the minimum oxygen concentrations can reach up to 4.19 mg.L^−1^, which is about 36% saturation (data from 2019). This level is unsatisfactory for the life of daphnids. For example, the standardized methodology for acute toxicity tests with *Daphnia magna* (ISO 6341:2012, 2012) recommends aerating samples before the assay starts when an oxygen concentration in them is lower than 40%, even if these tests are performed in open vessels in contrary to the DaphTox chamber, which is hermetically sealed. Therefore, the raw water was led first to a canister in which it was aerated, and only from this canister aerated water was sucked into the DaphTox chamber. This measure prevented the undesirable death of monitoring organisms caused by the low oxygen content in the monitored water.

### Pre-treatment of chlorinated drinking water

The process of treating raw water to produce drinking water can generate substances that can adversely affect water quality (Carmichael et al., 1982; Penders, 2011). That is why the monitoring of treated waters should be included in the early warning system.

In the case of ZWTP, it was possible to monitor the quality of produced drinking water only after its ozonation and chlorination. Because chlorine is highly toxic for monitoring organisms, the continuous dechlorination of the treated water was necessary for trouble-free continuous monitoring.

First, intensive aeration was applied to the treated water in a canister from which it was pumped into a chamber with monitoring organisms. However, this measure was not effective enough because the average residual chlorine concentration in the water was still 0.1 mg.L^−1^. That this concentration is toxic to the monitoring organisms was verified by experimental monitoring. Such observation was fully in line with the findings of other authors (Mattingley, 2017). *Daphnia magna* is the most sensitive to the total residual chlorine of the wide range of aquatic organisms investigated. For organisms, less than 24 h old (i.e. at the age at which they are deployed into the monitoring device), the concentration that causes 50% mortality of organisms at an exposure time of 48 h is 0.017 mg of total residual chlorine per litre.

Therefore, we had to look for a more effective way of dechlorination. We decided to use sodium thiosulphate (Na_2_S_2_O_3_) for the dechlorination of treated waters. This substance shows high efficiency in the dechlorination of aqueous solutions and at the same time has very low toxic effects on *Daphnia magna*. Its concentration up to 200 mg.L^−1^ does not have any negative effects on daphnids in acute toxicity tests (Basu & Dorner, 2010).

The substance was successfully used in the monitoring of waters treated by chlorination (Zeng et al., 2012). These authors continuously added to these waters a solution of sodium thiosulphate at a final concentration of 1.75 mg.L^−1^ in the monitored waters. The use of sodium thiosulphate for dechlorination of aqueous samples is also recommended by the Czech technical standard of water management (TNV 75 7768, 2006). This standard recommends the use of thiosulphate at a final concentration of 10 mg.L^−1^.

We performed our acute toxicity tests using the standardized methodology (ISO 6341:2012, 2012). The test solutions of Na_2_S_2_O_3_ were prepared in the final concentrations of 10 mg.L^−1^, 20 mg.L^−1^, 50 mg.L^−1^ and 100 mg.L^−1^. The potassium dichromate (K_2_Cr_2_O_7_) was used as a standard reference toxicant to assess the daphnids’ sensitivity. Its IC50 values for 24-h exposure ranged from 0.9 to 1.1 mg.L^−1^ and from 0.7 to 0.9 mg.L^−1^ for 48-h exposure.

At 48-h exposure, none of the investigated solutions of Na_2_S_2_O_3_ had adverse effects on *Daphnia magna*. Based on the results of ecotoxicological tests, we decided to use thiosulphate in a final concentration of 20 mg.L^−1^. The reason was the effort to achieve the maximum level of dechlorination. The effectivity of this procedure was verified by measuring when the content of total residual chlorine dropped to zero after the addition of thiosulphate to the waters after treatment. Test monitoring also proved the safety of treated waters after dechlorination for monitoring organisms.

The monitoring device (DaphTox) had to be supplemented with another synchronized peristaltic pump to be able to add the thiosulphate solution to the treated waters continuously. This measure allowed the device to suck equal volumes of monitored water and thiosulphate solution. These two media were combined into one supply hose by a connecting piece so that already treated water mixed with the thiosulphate solution flowed into the monitoring chamber of the device. This measure resulted in the elimination of a negative impact of chlorine-treated water on daphnids.

### Sample collection

If a significant reduction in the biological quality of water occurs during monitoring, the source of this problem must be determined. Therefore, it is necessary to take a water sample for detection analyses immediately. Because this situation can occur at any time, an automated sample collection is necessary.

A possibility to trigger automatic sample collection when the significant reduction of the biological quality of water is detected is not provided by either the manufacturer of the monitoring device or the manufacturer of the automatic sampler. Our technical solution, which is described in the utility model registered by the Industrial Property Office of the Czech Republic (Kabeláč & Racek, 2020), uses a pulse from the alarm relay of the monitoring device to start the automatic sampling of water. For this purpose, the relay outputs are connected to the sampler connectors. These inputs are configured by a closed circuit system. When the alarm relay on the monitoring device is switched on, the circuit closes and the sampling device responds to this signal by starting the automatic sampling according to the set sampling program.

### Increasing the sensitivity of monitoring

The possibility of increasing the sensitivity of monitoring was also tested. The aim was to detect the chronic impact of low concentrations of substances, which could be possibly present in monitored water. This problem was discussed by Penders (2011). He suggested increasing the sensitivity of online biological early warning systems for surface water quality control by the integration of solid-phase extraction of pollution from water samples. This process is very efficient but by its nature unsuitable for continuous use. We also consider it unsuitable for wide routine use because the concentration process is quite complex and requires additional relatively highly sophisticated equipment.

Our solution consists of extending the contact time of monitoring organisms with the monitored water. The device producer recommends a 7-day interval for the exchange of monitoring organisms. This is not only due to the need to carry out routine maintenance of the equipment (cleaning of pollution and biological growth in the water supply paths) but also due to the life cycle of daphnids.

In the case of the ZWTP, monitored water is relatively clean, so extending the interval of device maintenance is possible.

**Fig. 4:**
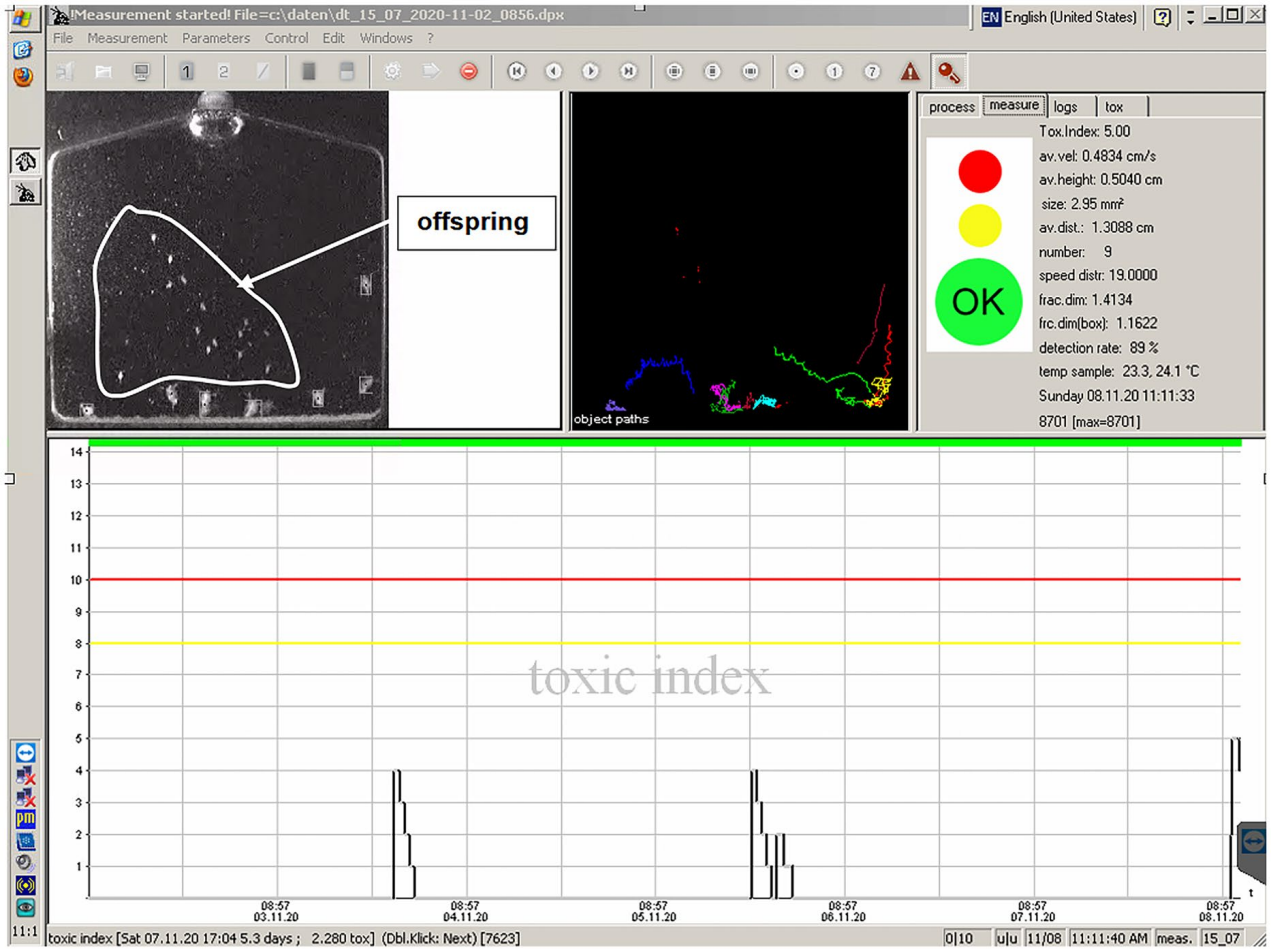
The situation with newborn organisms in the monitoring cell

Another possible problem with extending the contact time could be connected with the system of computer evaluation of the organisms’ reaction to monitored water quality. One of the important assessment parameters is the number of organisms in the chamber. At the beginning of the monitoring session, the start number of organisms is taken as a reference. Under optimal conditions, a parthenogenetic reproduction of mother organisms occurs after 8 to 10 days. This increases the number of organisms in the cell. However, because the software evaluation is adaptive and new reference limits can be set for the changing number of organisms in the chamber, so this fact did not prevent the implementation of prolonging the interval of exchange of monitoring organisms. Thus, it was possible to extend the exchange interval of monitoring organisms up to 30 days or more. Figure 4 shows the situation with newborn organisms. Recorded fluctuations in the curve at the bottom of the figure are associated with decreases in the locomotor activity of the parent organisms during offspring’s birth.

## Results and discussion

The main intention of the research was to suggest a new system of monitoring, which can guarantee a more reliable continuous control of the safe drinking water supply for the city of Prague and the surrounding area. The project aim also reflects the current security situation, when drinking water sources for large settlements may be threatened by criminal or terrorist activities. This situation is a significant change compared to the times when compliance with the principles set by the rules of protection zones of drinking water sources guaranteed relatively effective prevention of their unwanted pollution.

The implementation of continuous monitoring using DaphTox devices at the ZWTP represents a completely new approach to monitoring the biological quality of drinking water in the Czech Republic.

The newly proposed type of monitoring includes several new elements listed below.

*Daphnia magna* used as the monitoring organism is generally more sensitive to the possible negative effects of water pollution than routinely used trout. Its high sensitivity to cyanobacterial toxins (in contrast to trout) is also very advantageous when monitoring raw water from the drinking water reservoirs.

The assessment of the biological impact is observer-independent so the evaluation of monitoring organisms’ reactions is not subjectively influenced. Monitoring is automatically operated in real-time non-stop mode. The view of the actual situation and the special software evaluation of monitoring organisms’ condition is remotely accessible from anywhere with any device with a connection to the internet.

Automated collection of water samples started by alarm situation provides an immediate sample, which enables the determination of alarm causes more accurate.

The extension of the monitoring organism exchange interval, which was successfully tested, has moved the level of evaluation closer to the area of determining the possible chronic effects of water pollution. This measure contributes to increasing the sensitivity of monitoring.

Implementation of continuous monitoring of the biological quality of treated water makes the treatment plant’s early warning system more complex and thus the drinking water production safer.

Based on the facts mentioned above, it can be stated that the suggested new type of continuous biological monitoring can increase the efficiency and accuracy of the drinking water quality early warning system of the water treatment plant. In this way, the protective mechanisms of basic life resources for inhabitants of the city of Prague, of the Central Bohemian Region and the Vysočina Region can be strengthened.

## Conclusion

The test operation of the suggested type of monitoring of both types of water (raw and treated) lasts for more than 2 years. No cases of a significant reduction in the biological quality of the monitored waters were detected in the course of this test operation. During prolonged monitoring sessions, spontaneous reproduction of monitoring organisms in the chambers was noticed several times, so it can be stated that no chronic impact of both monitored water was recorded.

The operation of continuous biological monitoring with the use of DaphTox devices is more complicated compared to the use of trout for these needs. However, it is necessary to realize that the greater complexity of the suggested monitoring system is balanced by greater accuracy and operability of measurement. Currently, the monitoring is performed by a trained employee of the treatment plant with our technical assistance and consultations. This fact proved our assumption that even if the operation of the suggested monitoring system is more complicated, it is suitable for routine use.

## Data Availability

Data are available from the corresponding author on reasonable request.

## References

[CR1] Agrawal M, Yadav S, Patel C, Raipuria N, Agrawal MK (2012). Bioassay Methods to Identify the Presence of Cyanotoxins in Drinking Water Supplies and Their Removal Strategies.

[CR2] Basu OD, Dorner SM (2010). Potential aquatic health impacts of selected dechlorination chemicals. Water Quality Research Journal.

[CR3] Blanchette, M., & Haney, J. (2002). The effect of toxic *Microcystis aeruginosa* on four different populations of Daphnia. UNH Center for Freshwater Biology. Retrieved November 14, 2018, from https://scholars.unh.edu/cgi/viewcontent.cgi?article=1012&context=cfb

[CR4] Bownik A (2013). Effects of cyanobacterial toxins, microcystins on freshwater invertebrates. Polish Journal of Natural Science.

[CR5] Bratská, Z., & Riganová, N. (n.d.). Quality in the Starina reservoir. (in Slovak) Regionálny úrad verejného zdravotníctva so sídlom v Košiciach. Retrieved August 6, 2018, from https://www.smv.cz/res/archive/051/005760.pdf?seek=1429083269

[CR6] Carmi, O., & Theocharidou, M. (2019). Practical guidelines on requirements of continuous online water-quality monitoring system in drinking-water supply systems. Publications Office of the European Union. Retrieved February 10, 2020, from https://data.europa.eu/doi/10.2760/033873

[CR7] Carmichael NG, Winder C, Borges SH, Backhouse BL, Lewis PD (1982). Minireview: The health implications of water treatment with ozone. Life Sciences.

[CR8] Dunaj, J. (n.d.). Early warning system using biological monitoring in the production of drinking water at the Stakčín WTP. (in Slovak) Východoslovenská vodárenská spoločnosť a.s., Úpravňa vody Stakčín. Retrieved November 9, 2018, from http://wtwsk.2ka.cz/upload/files/BEWS__UV_Stakcin.pdf

[CR9] da Ferrão-Filho A, S., & Azevedo, S. M. F. O.  (2003). Effects of unicellular and colonial forms of toxic *Microcystis aeruginosa* from laboratory cultures and natural populations on tropical cladocerans. Aquatic Ecology.

[CR10] Gunatilaka, A., & Diehl, P. (2001). A brief review of chemical and biological continuous monitoring of rivers in Europe and Asia. In F. M. Butterworth, A. Gunatilaka, & M. E. Gonsebatt (Eds.), Biomonitors and biomarkers as indicators of environmental change 2: A handbook (pp. 9–28). Boston, MA: Springer US. 10.1007/978-1-4615-1305-6_2

[CR11] Hasan, J., Goldbloom-Helzner, D., Ichida, A., Rouse, T., & Gibson, M. (2005, August 25). Technologies and techniques for early warning systems to monitor and evaluate drinking water quality: A state-of-the-art review. U.S. Environmental Protection Agency, Office of Water, Office of Science and Technology, Washington DC,20460. Retrieved July 20, 2018, from https://cfpub.epa.gov/si/si_public_record_report.cfm?Lab=NHSRC&address=nhsrc/&dirEntryId=144729

[CR12] Herrera NA, Echeverri LF, Ferrão-Filho AS (2015). Effects of phytoplankton extracts containing the toxin microcystin-LR on the survival and reproduction of cladocerans. Toxicon.

[CR13] ISO 6341:2012. (2012). Water quality — Determination of the inhibition of the mobility of *Daphnia magna* Straus (Cladocera, Crustacea) — Acute toxicity test

[CR14] Kabeláč R, Racek J (2020). Equipment for the sampling of watercourses. Utility Model.

[CR15] Lechelt M, Blohm W, Kirschneit B, Pfeiffer M, Gresens E, Liley J (2000). Monitoring of surface water by ultrasensitive Daphnia toximeter. Environmental Toxicology.

[CR16] Liška, M., Soukupová, K., Dobiáš, J., Metelková, A., Goldbach, J., & Kvítek, T. (2016). Water quality in drinking water reservoir Švihov on Želivka river and its river basin, with focus on specific organic compounds. (in Czech) *Vodohospodářské technicko-ekonomické informace*, *58*(3), 4–11. 10.46555/VTEI.2016.03.001

[CR17] Mattingley, L. (2017). The impact of chlorine and chlorinated compounds in freshwater systems. Salmon & Trout Conservation, 7 pp.. Retrieved July 25, 2018, from https://www.salmon-trout.org/wp-content/uploads/2017/09/STC-The-impact-of-chlorine-and-chlorinated-compounds-in-freshwater-systems.pdf

[CR18] Mičaník, T., Sýkora, F., Chrastina, D., Cielecká, N., Kucharczyková, V., Kristová, A., et al. (2020). Space-time dynamic of pesticide loading in the drinking water reservoir Švihov. (in Czech) *Vodohospodářské technicko-ekonomické informace,**62*(2), 6–16. 10.46555/VTEI.2020.02.004

[CR19] Penders, E. J. M. (2011). Development of aquatic biomonitoring models for surface waters used for drinking water supply. Wageningen University, Wageningen. Retrieved September 17, 2018 from https://edepot.wur.nl/185117

[CR20] Randák, T., Žlábek, V., Turek, J., Velíšek, J., & Kolářová, J. (2011). Use of rainbow trout (*Oncorhynchus mykiss*) for ecotoxicological monitoring of water quality. (in Czech), *Edice metodik (technologická řada)*, 111, FROV JU, Vodňany.

[CR21] Schmidt JR, Wilhelm SW, Boyer GL (2014). The fate of microcystins in the environment and challenges for monitoring. Toxins.

[CR22] Soldán P (2011). Possible way to substantial improvement of early warning system in the International Odra (Oder) River Basin. Environmental Monitoring and Assessment.

[CR23] Storey MV, van der Gaag B, Burns BP (2011). Advances in on-line drinking water quality monitoring and early warning systems. Water Research.

[CR24] TNV 75 7768. (2006). Water Quality - Evaluation of the efficiency of industrial wastewater treatment by toxicological determination. (in Czech)

[CR25] Tušil, P. (2016). The water treatment plant in Želivka – SWOT analysis of reconstruction and modernization. (in Czech) *Vodohospodářské technicko-ekonomické informace*, *58*(2), 28–37. 10.46555/VTEI.2016.01.004

[CR26] Vasconcelos, V. (2001). Cyanobacteria toxins: Diversity and ecological effects. *Limnetica*, *20*(1), 45–58. Retrieved June 11, 2018, from https://www.limnetica.com/documentos/limnetica/limnetica-20-1-p-45.pdf

[CR27] Zanchett G, Oliveira-Filho EC (2013). Cyanobacteria and cyanotoxins: From impacts on aquatic ecosystems and human health to anticarcinogenic effects. Toxins.

[CR28] Zeng Y, Fu X, Ren Z (2012). The effects of residual chlorine on the behavioural responses of *Daphnia magna* in the early warning of drinking water accidental events. Procedia Environmental Sciences.

